# Pan-Cancer Analysis Identified CD93 as a Valuable Biomarker for Predicting Patient Prognosis and Immunotherapy Response

**DOI:** 10.3389/fmolb.2021.793445

**Published:** 2022-02-21

**Authors:** Wen Tong, Guangyu Wang, Liuyang Zhu, Yi Bai, Zirong Liu, Long Yang, Hao Wu, Tao Cui, Yamin Zhang

**Affiliations:** ^1^ Tianjin First Central Hospital Clinic Institute, Tianjin Medical University, Tianjin, China; ^2^ Department of Hepatobiliary Surgery, Tianjin First Central Hospital, School of Medicine, Nankai University, Tianjin, China; ^3^ State Key Laboratory of Drug Delivery Technology and Pharmacokinetics, Tianjin Institute of Pharmaceutical Research, Tianjin, China; ^4^ Research Unit for Drug Metabolism, Chinese Academy of Medical Sciences, Beijing, China

**Keywords:** CD93, biomarker, immunotherapy, pan-cancer, prognosis, immune infiltration

## Abstract

**Background:** The rapid development of immunotherapy has significantly improved patient outcomes in recent years. CD93, a novel biomarker expressed on vascular endothelial cells, is essential for tumor angiogenesis. Recent studies have shown that CD93 is closely related to immune cell infiltration and immunotherapy. However, its role in pan-cancer has not been reported.

**Methods:** The Cancer Genome Atlas (TCGA), Human Protein Atlas (HPA), cbioportal, Gene Expression Omnibus (GEO), Tumor Immune Estimation Resource (TIMER2.0), and the Tumor–Immune System Interactions and Drug Bank (TISIDB) databases were used to analyze CD93 in pan-cancers. R software was used for statistical analysis and mapping.

**Results:** There were significant differences in the expression of CD93 between tumor tissues and adjacent normal tissues in pan-cancer. The high expression of CD93 was associated with poor prognosis and high TNM stage in multiple tumor types. However, a high expression of CD93 was a protective factor in kidney renal clear cell carcinoma (KIRC). In addition, CD93 was closely related to immune cell infiltration in tumor tissues. Moreover, CD93 presented a robust correlation with immune modulators and immunotherapeutic markers [e.g., tumor mutation burden (TMB) and microsatellite instability (MSI)]. The results of gene set enrichment analysis (GSEA) showed that CD93 was correlated with tumor angiogenesis. Importantly, patients with a low expression of CD93 were more sensitive to immunotherapy in urothelial cancer.

**Conclusion:** CD93, which is involved in various immune responses, controls immune cell infiltration and impacts on the malignant properties of various cancer types. Therefore, CD93 has potential value to be biomarker for determining the prognosis and immune infiltration in multiple cancers.

## Introduction

The latest research revealed that tumor angiogenesis involved a series of complex processes, including the regulation of endothelial cell migration and extracellular matrix deposition (Liao et al., 2020; Mao et al., 2020; Unterleuthner et al., 2020). Endothelial cell migration is essential to angiogenesis, enabling the outgrowth of new blood vessels both in physiological and pathological contexts. Growing evidence indicated that CD93 plays an important regulatory role in tumor angiogenesis (Lugano et al., 2018; Barbera et al., 2021). In addition, CD93 was highly expressed in vascular endothelial cells of tumor tissues, but weakly expressed in non-proliferative vascular endothelial cells (Sun et al., 2021).

Multimerin 2 (MMRN2) and CD93 are co-expressed in many kinds of tumors. MMRN2 is a type of pan-endothelial extracellular matrix protein that can be used as a specific ligand of CD93. The interaction of CD93 and MMRN2 can promote endothelial cell adhesion and migration, thus promoting pathological angiogenesis (Galvagni et al., 2017). Furthermore, CD93 could promote β1 integrin activation and fibronectin fibrillogenesis, thus performing a significant role in vascular maturation and formation of the extracellular matrix during tumor angiogenesis (Lugano et al., 2018). Recently, studies have shown that CD93 controls the migration of endothelial cells by activating the small GTPase of Rho family (Barbera et al., 2019). Migration requires the activation of a variety of signaling pathways, and their elucidation will increase the opportunity to developing new drugs for anti-angiogenic therapy. In addition, CD93 plays an important role in innate immunity. Recent studies have shown that CD93 is a member of the lectin XIV group with the C-type lectin domain (CTLD). CD93 can interact with CpG motifs and act as a new receptor to transfer bacterial DNA to endosomal Toll-like receptor 9 (TLR9) (Nativel et al., 2019).

Furthermore, CD93 mediates the enhancement of phagocytosis in monocytes and macrophages upon interaction with soluble defense collagens and plays a role in intercellular adhesion (Nativel et al., 2019). Meanwhile, CD93 is an important neuroimmunomodulatory factor in the control of central nervous system inflammation (Griffiths et al., 2018).

Clinical studies have shown that the high expression of CD93 was closely related to the poor effects of immunotherapy in patients. IGFBP7/CD93 overexpression was associated with poor treatment response in cancer patients treated with anti-PD1/PDL1 (Sun et al., 2021). Furthermore, animal experiments revealed that CD93 blockers in mice promoted drug delivery, thus improving the antitumor response to gemcitabine or fluorouracil (Sun et al., 2021). In addition, the blockage of the CD93 pathway leads to a large increase of intratumoral effector T cells, which makes mouse tumors sensitive to immune checkpoint therapy (Sun et al., 2021).

To the best of our knowledge, this is the first study focusing on the value of CD93 in pan-cancer. The relationship between tumor mutation load (tumor mutation burden, TMB), microsatellite instability (MSI), and CD93 expression was studied in this research. In addition, the correlation between the expression and mutation of CD93 and the effect of immunotherapy was investigated in an external verification dataset. Besides, this research revealed the relationship between CD93 expression and immune cell infiltration and immune biomarkers, thus providing valuable insight into the role of CD93 in cancer immunotherapy. We believe that this study lays a solid foundation for further exploration of the value of CD93 in cancer prognostic biomarkers and immunotherapy in the future.

## Materials and Methods

### Data Collection

Transcriptome data, somatic mutation data, and clinical information of 33 pan-cancer types were downloaded from the UCSC Xena platform (https://xena.ucsc.edu/) (Goldman et al., 2020). The abbreviations and full names of the 33 tumor types are shown in Table 1. Moreover, data on metastatic melanoma treated with pembrolizumab (GSE78220) and renal cell carcinoma treated with nivolumab (GSE67501) were acquired from the Gene Expression Omnibus (GEO) database (https://www.ncbi.nlm.nih.gov/geo/). In addition, from previously published studies, we obtained data on urothelial cancer treated with atezolizumab (Mariathasan et al., 2018).

**TABLE 1:** Abbreviations and details of the 33 cancer types used in this study.

Abbreviation	Detail
ACC	Adrenocortical carcinoma
BLCA	Bladder urothelial carcinoma
BRCA	Breast invasive carcinoma
CESC	Cervical squamous cell carcinoma and endocervical adenocarcinoma
CHOL	Cholangiocarcinoma
COAD	Colon adenocarcinoma
DLBC	Lymphoid neoplasm diffuse large B-cell lymphoma
ESCA	Esophageal carcinoma
GBM	Glioblastoma multiforme
HNSC	Head and neck squamous cell carcinoma
KICH	Kidney chromophobe
KIRC	Kidney renal clear cell carcinoma
KIRP	Kidney renal papillary cell carcinoma
LAML	Acute myeloid leukemia
LGG	Brain lower grade glioma
LIHC	Liver hepatocellular carcinoma
LUAD	Lung adenocarcinoma
LUSC	Lung squamous cell carcinoma
MESO	Mesothelioma
OV	Ovarian serous cystadenocarcinoma
PAAD	Pancreatic adenocarcinoma
PCPG	Pheochromocytoma and paraganglioma
PRAD	Prostate adenocarcinoma
READ	Rectum adenocarcinoma
SARC	Sarcoma
SKCM	Skin cutaneous melanoma
STAD	Stomach adenocarcinoma
TGCT	Testicular germ cell tumors
THCA	Thyroid carcinoma
THYM	Thymoma
UCEC	Uterine corpus endometrial carcinoma
UCS	Uterine carcinosarcoma
UVM	Uveal melanoma

### CD93 Expression Profiles

Based on the website of the Human Protein Atlas (HPA; http://www.proteinatlas.org/), we explored the messenger RNA (mRNA) and protein levels of CD93 in various cancer tissues and normal tissues. Meanwhile, we downloaded the immunohistochemical images of the CD93 protein in multiple cancer and normal tissues from this website. The association between the expression of CD93 and other clinical characteristics (age, gender, and TNM stage) was also investigated. Meanwhile, the CD93 expression activity between normal and tumor tissues was analyzed *via* the R package (version 4.0.3) of GSEABase and gene set variation analysis (GSVA) (Liu et al., 2021a; Liu et al., 2021d).

### Association Between CD93 Expression and Prognosis

Furthermore, by using the R survival package, we analyzed the prognostic value of CD93 in pan-cancer *via* univariate Cox regression analysis. The considered survival outcomes included overall survival (OS), disease-free survival (DFS), disease-specific survival (DSS), and progression-free survival (PFS). A hazard ratio (HR) >1 indicates that a high expression of CD93 is a high-risk factor in a cancer species; otherwise, it is a protective factor. The results were visualized with the “forestplot” package in R.

### Immune Infiltration and Immunotherapy

The ESTIMATE package was used to calculate the tumor purity in 33 cancer types (Liu et al., 2021b; Liu et al., 2021c). Specifically, the ESTIMATE score is the sum of the immune score (representing the immune component) and the stromal score (representing the stromal component), which indirectly represents tumor purity (Yoshihara et al., 2013). The higher the immune and stromal scores, the higher the content of immune and stromal components in the tumor tissue, which will lead to lower tumor purity. Furthermore, the abundance of immune cell in tumor tissues was estimated using the CIBERSORT algorithm (Newman et al., 2015; Liu et al., 2021e). In addition, we investigated the correlation between CD93 expression and TMB and MSI. The results were shown as a radar plot.

Next, the underlying relationship between the expression of CD93 and three types of immune-related biomarkers, namely, immune inhibitors, immune stimulators, and major histocompatibility complex (MHC) molecules, was investigated through the Tumor–Immune System Interactions and Drug Bank (TISIDB) database (http://cis.hku.hk/TISIDB/) (Ru et al., 2019). We showed four immune-related genes that were most correlated with the expression of CD93 in each figure. Three independent external cohorts (GSE78220, GSE67501, and IMvigor210) were selected to evaluate the relationship between the expression of CD93 and immunotherapy. The correlation between the expression of CD93 and the immune subtype and response to immunotherapy was also investigated through the TISIDB database.

### Gene Set Enrichment Analysis

Finally, based on the Gene Ontology (GO) database, gene set enrichment analysis (GSEA) was performed to investigate the differential pathways among the low and high CD93 expression groups. The pathways with the top five highest normalized enrichment scores and *p*-value <0.05 were considered and presented in plots.

## Results

### CD93 Expression Profile in Human Normal and Tumor Tissues

**FIGURE 1:**
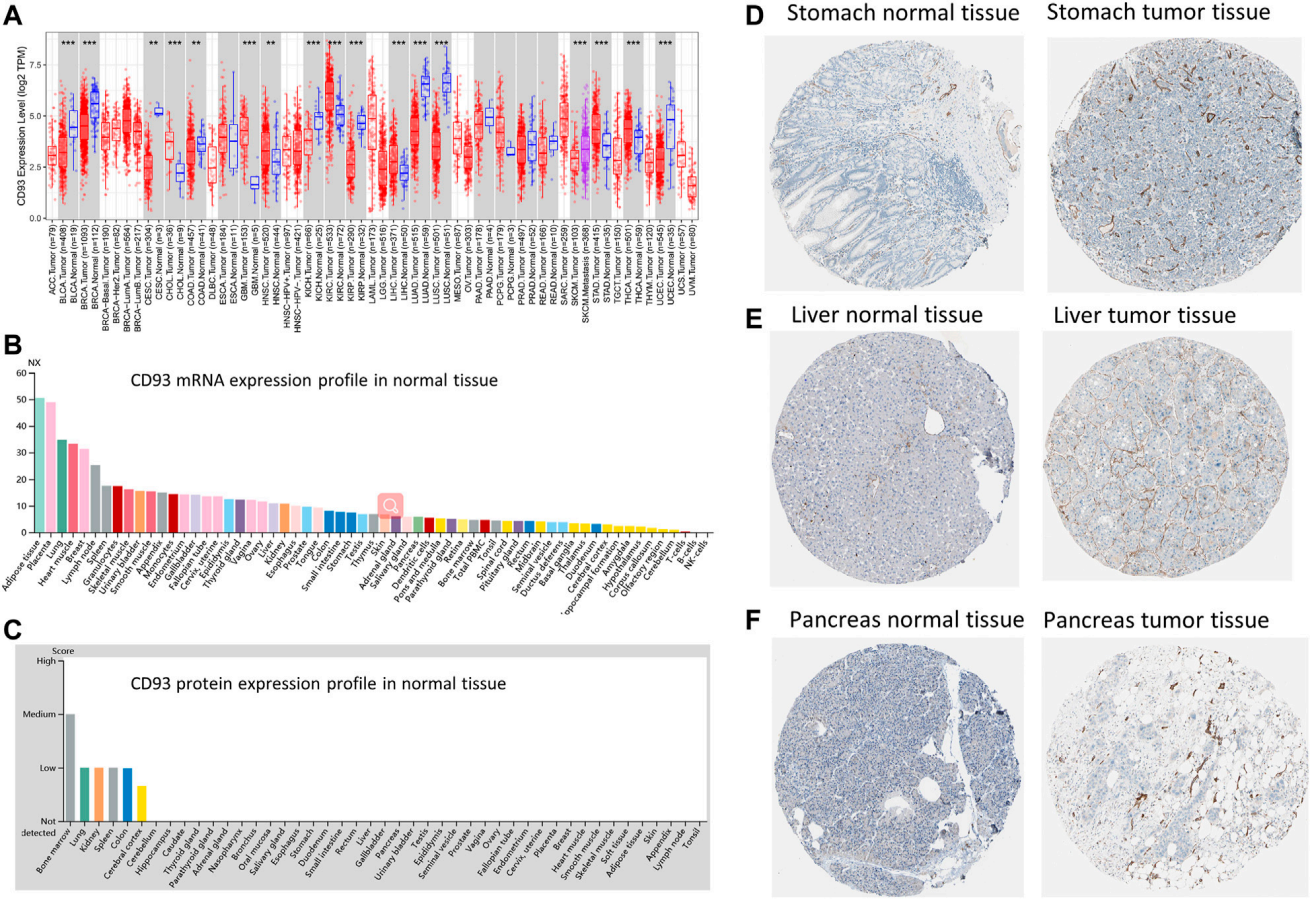
Gene and protein expression profiles of CD93 in tumor and normal tissues. **(A)** Differential expression of CD93 between normal and tumor tissues based on the Tumor Immune Estimation Resource (TIMER2.0) database. *Red*, *blue*, and *magenta* represent tumor, normal, and metastasis tissues, respectively. **(B)** CD93 mRNA expression overview in normal human tissues. **(C)** CD93 protein expression overview in human normal tissues. **(D–F)** Representative immunohistochemistry (IHC) images of CD93 proteins in normal and tumor tissues of the breast, liver, and pancreas.

We used the Tumor Immune Estimation Resource (TIMER2.0) database to explore the expression of CD93 in pan-cancer. In Figure 1A, the expression level of CD93 was significantly upregulated in six cancer types (i.e., CHOL, GBM, KIRC, LIHC, STAD, and THCA; all *p* < 0.01) and downregulated in 10 cancer types (i.e., BLCA, BRCA, CESC, COAD, HNSC, KICH, KIRP, LUAD, LUSC, and UCEC; all *p* < 0.01) when compared with corresponding normal tissues. Furthermore, a significantly higher CD93 expression was observed in SKCM metastatic tissues compared with SKCM tissues (*p* < 0.01; Figure 1A). The mRNA and protein levels of CD93 in multiple cancer types were also explored *via* the HPA dataset. According to Figure 1B, the CD93 mRNA was highly expressed in adipose and placenta tissues. Subsequently, we explored the protein level of CD93 and found it to be negative in most normal tissues (Figure 1C). Representative immunohistochemistry (IHC) images displayed that the CD93 protein was mostly enriched in membranes and had low expression in normal tissues when compared with tumor tissues in the stomach, liver, and pancreas (Figures 1D–F, respectively).

### Correlation Between CD93 Expression and Patient’s Prognosis

**FIGURE 2:**
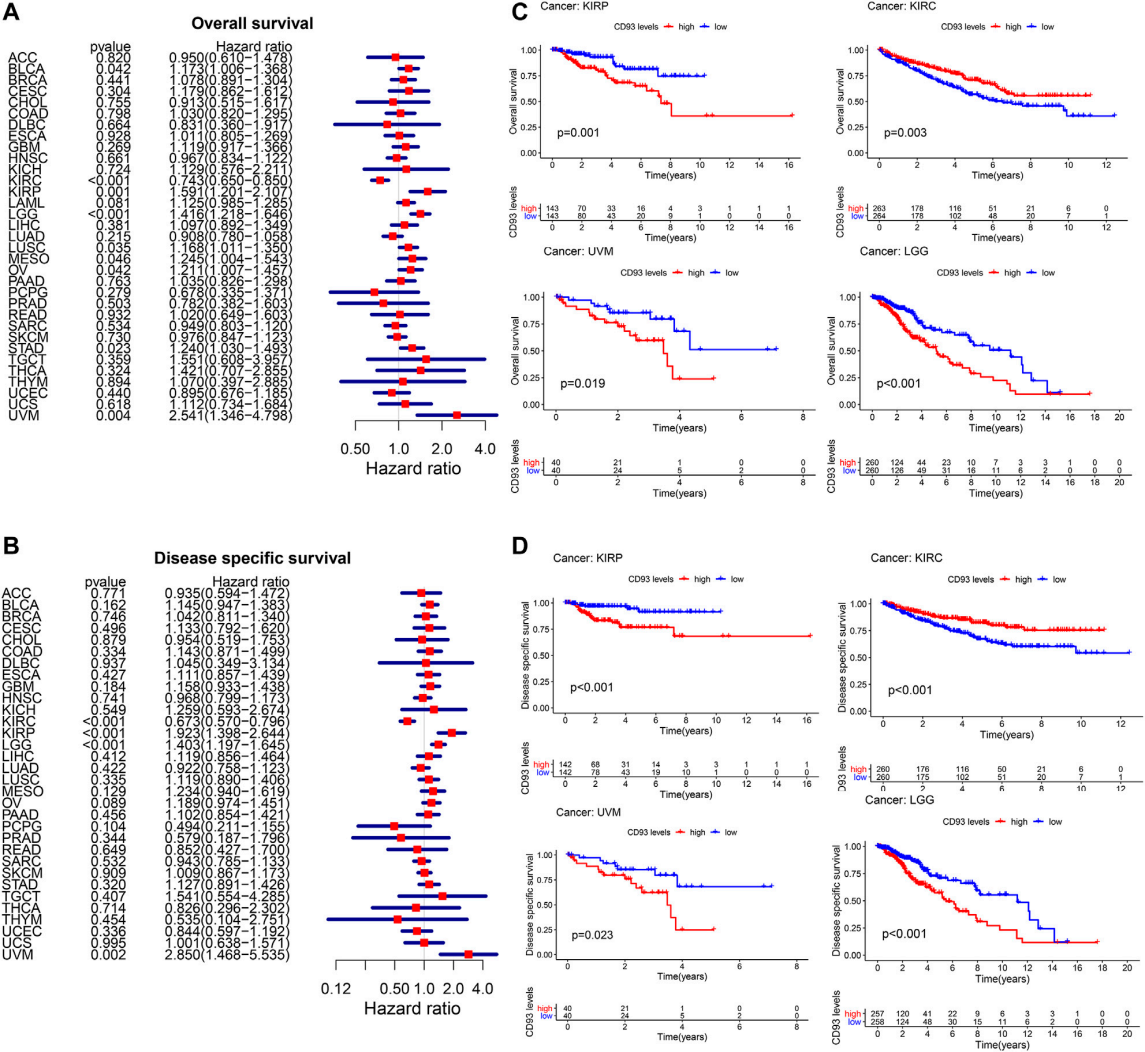
Relationship between CD93 expression and patient prognosis. **(A,B)** Correlation between the expression of CD93 and overall survival (OS) and disease-specific survival (DSS) in multiple tumor types based on The Cancer Genome Atlas (TCGA). **(C)** Difference in the OS between the CD93 high and low expression groups in four cancer types (KIRP, KIRC, UVM, and LGG). **(D)** Difference in the DSS between the CD93 high and low expression groups in four cancer types (KIRP, KIRC, UVM, and LGG).

We obtained the RNA sequences and clinical data from The Cancer Genome Atlas (TCGA) and UCSC Xena, respectively, and analyzed the prognostic value of CD93 in pan-cancer. According to Figure 2A, an elevated CD93 expression was significantly associated with poorer OS in KIRP (HR = 1.59, *p* = 0.001), UVM (HR = 2.54, *p* = 0.004), LGG (HR = 1.41, *p* < 0.001), STAD (HR = 1.24, *p* = 0.02), LUSC (HR = 1.16, *p* = 0.03), BLCA (HR = 1.17, *p* = 0.04), OV (HR = 1.21, *p* = 0.04), and MESO (HR = 1.24, *p* = 0.04) and better OS in KIRC (HR = 0.74, *p* < 0.001). Survival analysis showed that OS was significantly correlated with CD93 expression. The four most relevant cancers are displayed in Figure 2C. Moreover, DSS analysis was performed to exclude potential factors that interfered with survival. For example, patients who died from causes other than the disease being studied were not counted. The results of the analysis on DSS (Figure 2B) were similar to those of OS and showed that an elevated CD93 expression was significantly associated with poorer DSS in KIRP (HR = 1.92, *p* < 0.001), LGG (HR = 1.40, *p* < 0.001), and UVM (HR = 2.85, *p* = 0.002) and better DSS in KIRC (HR = 0.67, *p* < 0.001). The DSS curves are shown in Figure 2D (*p* < 0.05). In terms of DFS, a significant negative association was found in KIRP (Supplementary Figures S1A,B). Meanwhile, the analysis on PFS also demonstrated that CD93 overexpression was a protective factor in KIRC, but a risk factor in KIRP, LGG, and UVM (*p* < 0.05; Supplementary Figures S1C,D).

### Relationship Between CD93 Expression and Clinical Character

**FIGURE 3:**
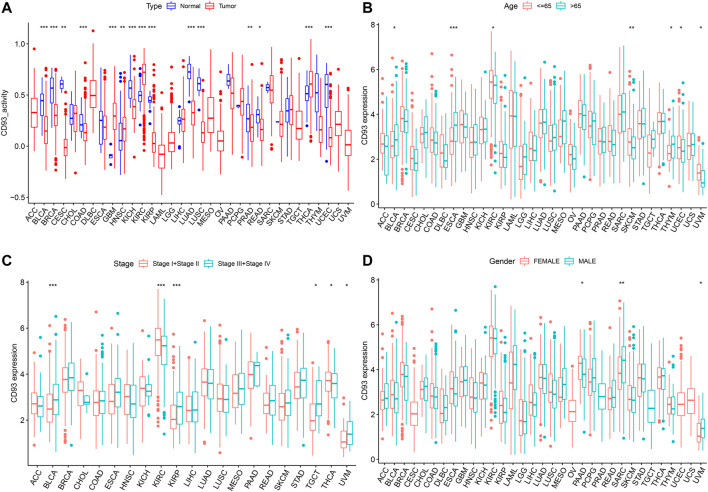
Relationship between the expression level of CD93 and clinical characteristics. **(A)** Differential analysis of CD93 expression activity between cancer tissue and adjacent normal tissue. **(B–D)** Correlation between age, tumor stage, and gender with CD93 expression. *p < 0.05, **p < 0.01, ***p < 0.001.

As shown in Figure 3A, the activity of CD93 was significantly upregulated in tumor tissues of GBM, HNSC, KIRC, and THCA, but downregulated in tumor tissues of CESC, KIRP, LUAD, KICH, LUSC, BRCA, PRAD, BLCA, PEAD, COAD, and UCEC. When compared with young patients, CD93 was highly expressed in BLCA, ESCA, and THYM, but weakly expressed in KIRC, SKCM, UCEC, and UVM (*p* < 0.01; Figure 3B). Meanwhile, patients with a high CD93 mRNA level were associated with advanced tumor stage in BLCA and KIRP (*p* < 0.001; Figure 3C). On the contrary, the expression of CD93 was higher in lower tumor stages than in higher tumor stages in KIRC (*p* < 0.001; Figure 3C). Interestingly, the results indicated significant gender-based differences in the CD93 expression of PAAD, SARC, and UVM. The expression of CD93 was higher in females with PAAD than in males, but the results were the opposite in SARC and UVM (*p* < 0.05; Figure 3D).

### Pan-Cancer Analysis of the Association of CD93 With Tumor Immunity

**FIGURE 4:**
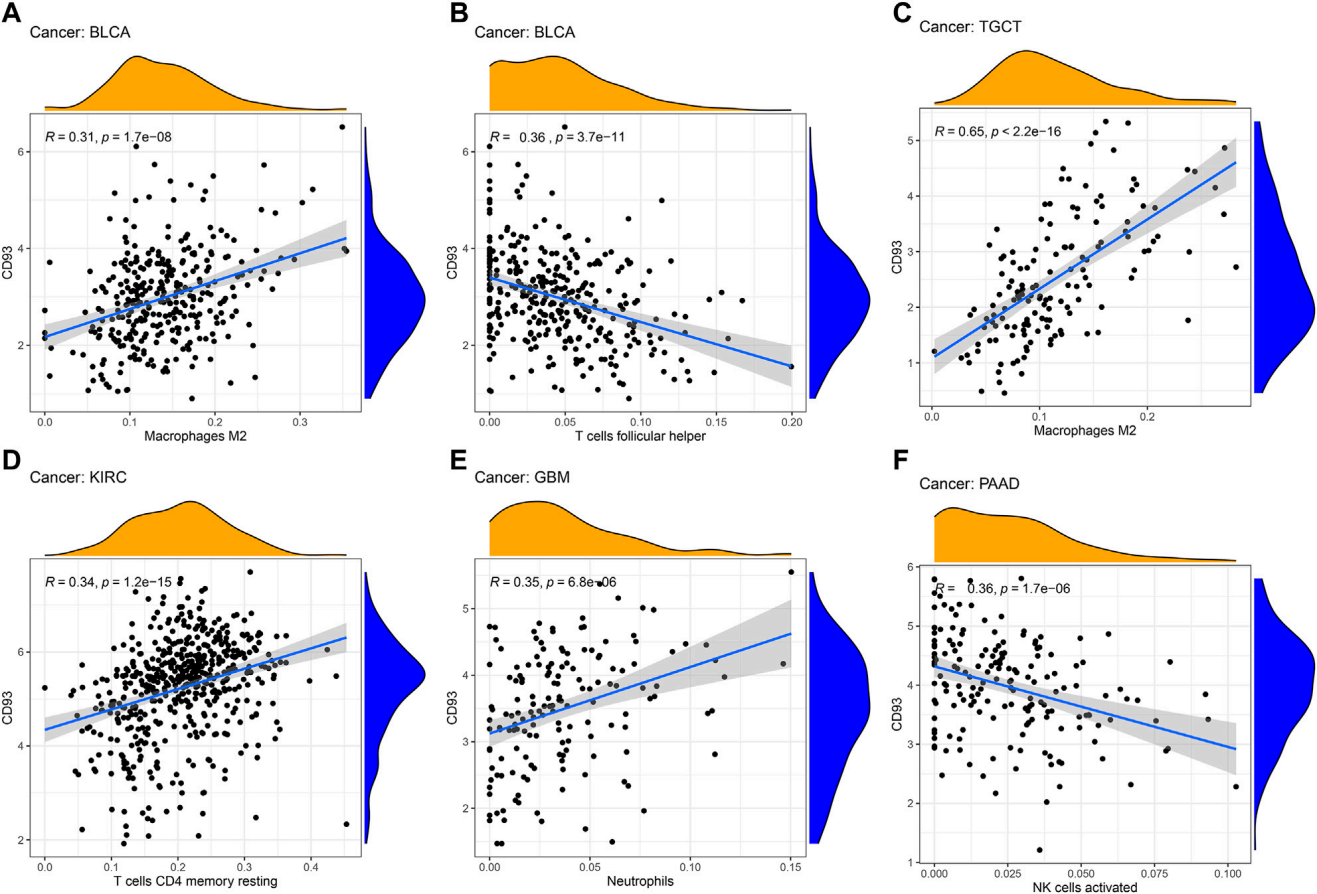
Correlation between CD93 expression and immune cell infiltration. **(A–F)**: The correlation analysis between CD93 expression and immune cell infiltration in BLCA, TGCT, KIRC, GBM and PAAD.

Since the expression of CD93 was closely associated with survival in KIRP, KIRC, LGG, and UVM, we further analyzed the relationship between the immune-related score and the expression of CD93. The stromal score, immune score, and ESTIMATE score are summarized in Supplementary Figures S2A–I (*p* < 0.01, |*R*| > 0.4). It was obvious that the expression level of CD93 was positively associated with the stromal score, immune score, and ESTIMATE score in KIRP, LGG, and UVM. In addition, we also explored the correlation of immune cell infiltration with CD93 expression (Figures 4A–F).

**FIGURE 5:**
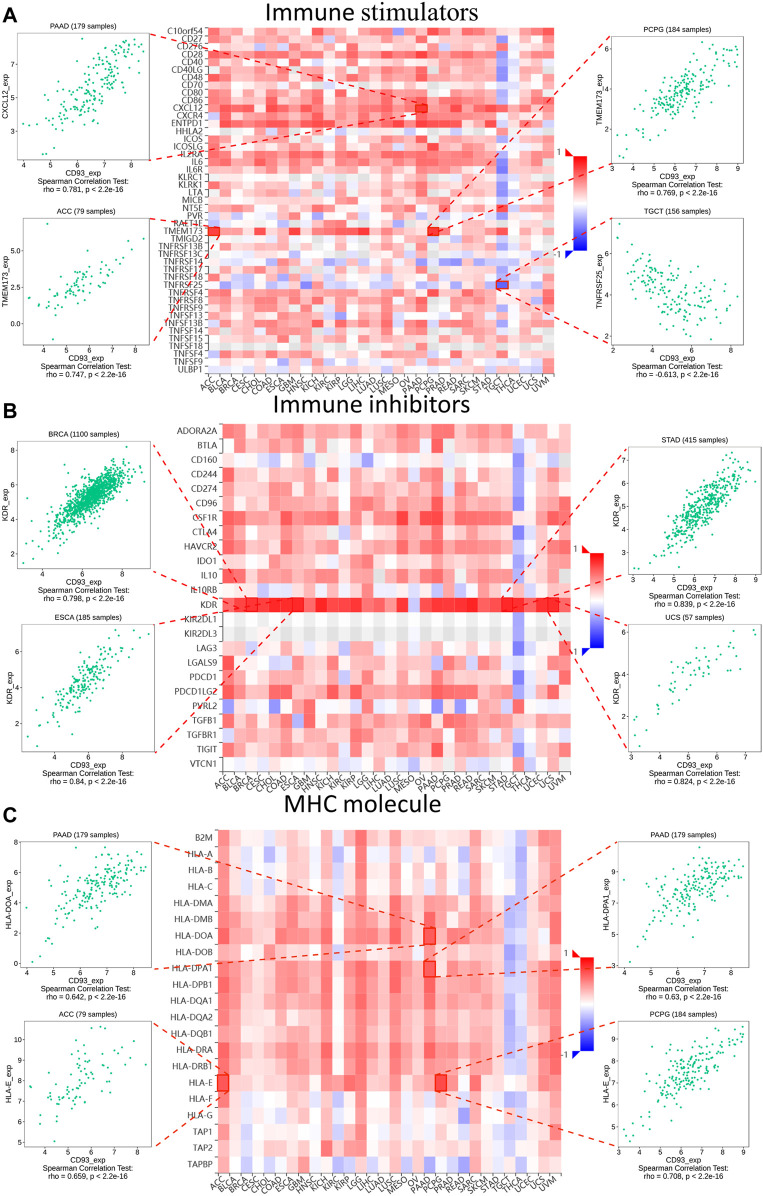
Relationship between three kinds of immunomodulators and CD93 expression according to the Tumor–Immune System Interactions and Drug Bank (TISIDB) database. Expression correlations between CD93 and immune stimulators **(A)**, immune inhibitors **(B)**, and MHC molecules **(C)**. *Red* and *blue* represent positive and negative correlations, respectively. The four immune genes most strongly associated with CD93 expression are shown in the dot plot.

Subsequently, the relationship between the expression of CD93 and three different immune modulators was explored *via* the TISIDB dataset. As shown in Figure 5A, 45 types of immune stimulators have been analyzed. The expression of CD93 was positively correlated with *CXCL12* in PAAD and *TMEM173* in ACC and PCPG, whereas it showed a negative relation with *TNFRSF25* in TGCT. Meanwhile, KDR, as one of the 24 immune inhibitors (Figure 5B), had a significant positive correlation with the expression of CD93 in BRCA, ESCA, STAD, and UCS. Furthermore, as illustrated in Figure 5C, the expression of CD93 was positively associated with *HLA-DOA* (major histocompatibility complex, class II, DO alpha) and *HLA-DPA1* (major histocompatibility complex, class II, DP alpha 1) in PAAD, as well as *HLA-E* (major histocompatibility complex, class I, E) in ACC and PCPG.

**FIGURE 6:**
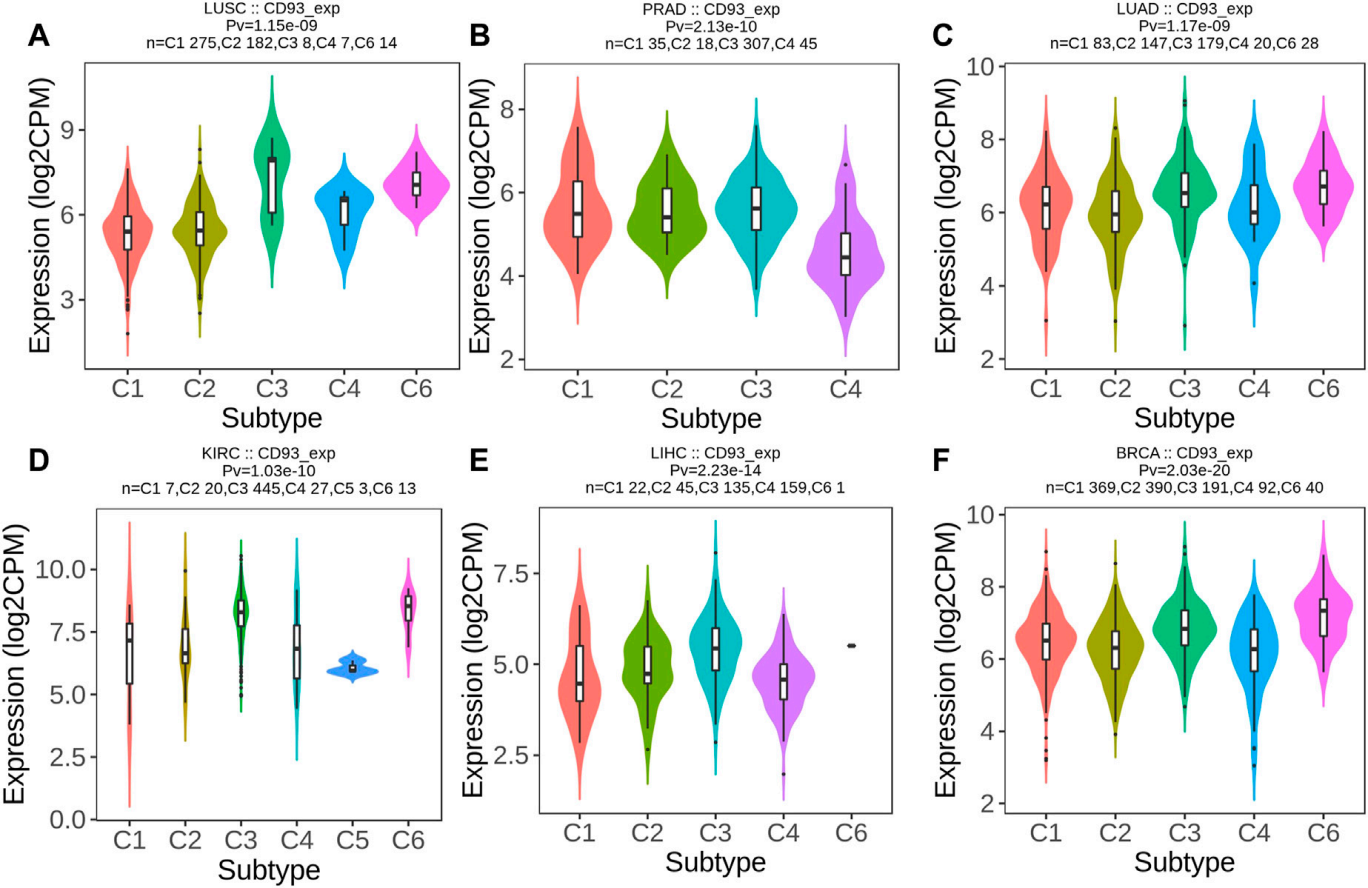
CD93 expression and immune subtypes according to the Tumor–Immune System Interactions and Drug Bank (TISIDB) database. **(A–F)** Expression level of CD93 among different immune subtypes in six cancer types (LUSC, PRAD, LUAD, KIRC, LIHC, and BRCA).

**FIGURE 7:**
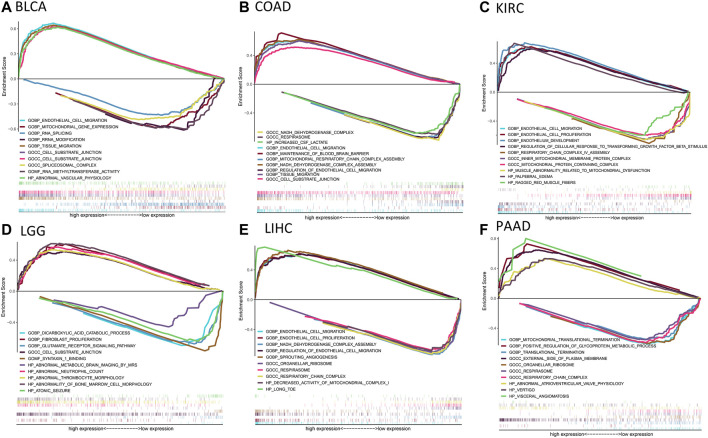
Results of gene set enrichment analysis (GSEA) based on the Gene Ontology (GO) database. **(A–F)**: The GSEA analysis between CD93 high and low expression group in BLCA, COAD, KIRC, LGG, LIHC and PAAD. The *left* and *right* sides of each panel represent the enriched pathways of the CD93 high and low expression group, respectively.

Then, we used the TISIDB database to explore whether CD93 was correlated with the immune subtype in multiple cancer types. All tumor samples in the TISIDB database were divided into six immune subtypes: C1, wound healing; C2, IFN-gamma dominant; C3, inflammatory; C4, lymphocyte depleted; C5, immunologically quiet; and C6, TGF-b dominant. Specifically, the expression of CD93 was significantly correlated with the immune subtypes in LUSC, PRAD, LUAD, KIRC, LIHC, and BRCA (all *p* < 0.01; Figures 6A–F). In order to further clarify the biological function of CD93, we explored in which functions the gene set was enriched between the high and low expression groups of CD93 based on the GO database. As shown in Figures 7A–F, we found that, in COAD, BLCA, KIRC, and LIHC, the gene set of the CD93 high expression group was mainly enriched in the endothelial cell migration and tissue migration pathway, which may be closely related to tumor angiogenesis.

### The Genetic Alteration Landscape of CD93 in Pan-Cancer

**FIGURE 8:**
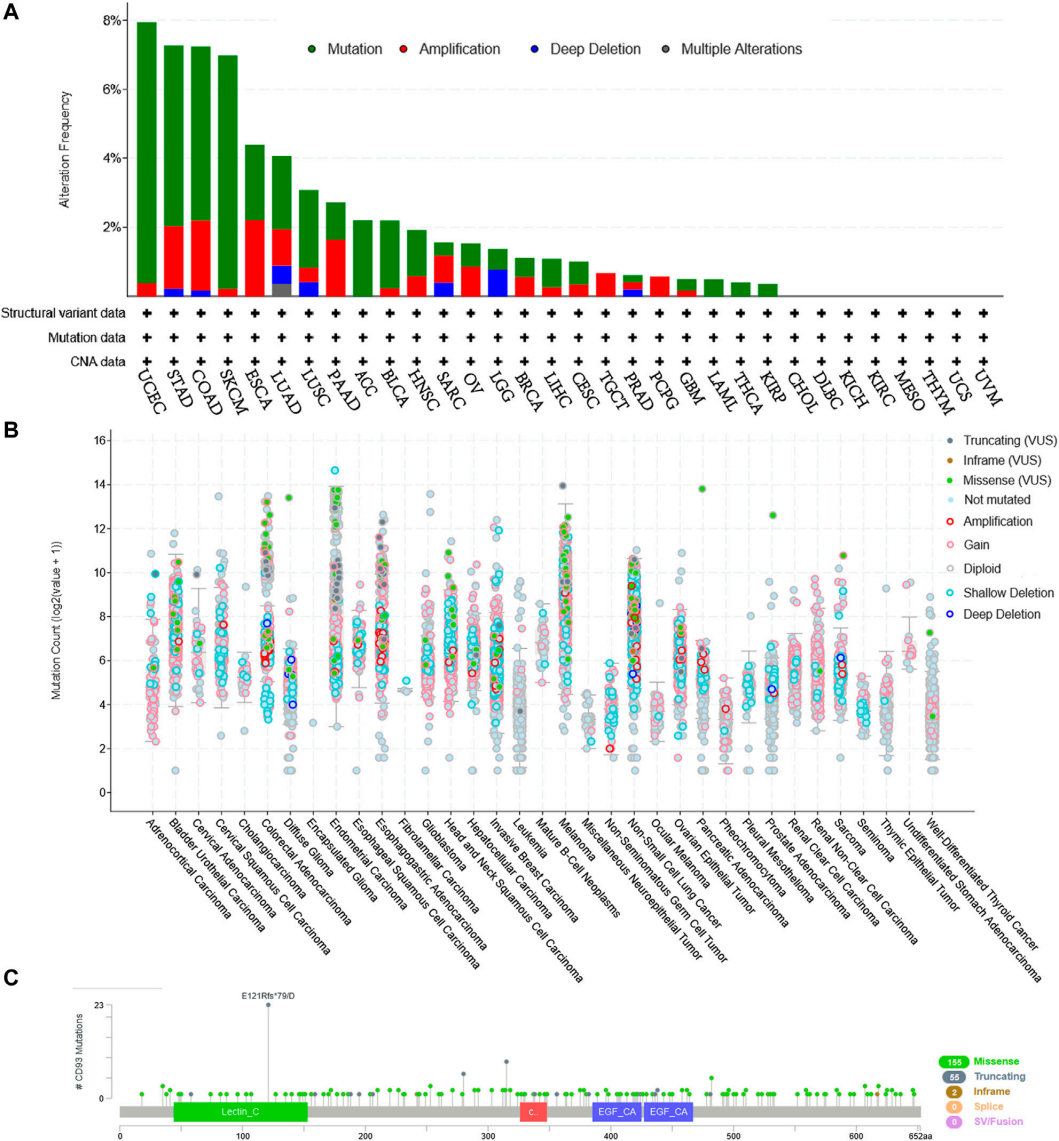
CD93 mutation landscape according to the cBioPortal database. **(A)** CD93 mutation frequency in multiple cancer types in The Cancer Genome Atlas (TCGA). **(B)** Mutation counts across different cancers. **(C)** Mutation diagram of CD93 in different cancer types across protein domains.

Based on the cBioPortal database, we analyzed the genetic alteration of CD93 in pan-cancer. As shown in Figures 8A,B, the top three tumors with the highest frequency of CD93 mutation were UCEC, STAD, and COAD, among which the most common genetic alteration was gene mutation. Moreover, gene mutation of CD93 was the only genetic alteration type in ACC, LAML, THCA, and KIRP. In addition, genetic amplification had an alteration frequency second only to gene mutation and was the only genetic alteration type in PCPG. Furthermore, a total of 212 mutation sites (including 155 missense, 55 truncating, 2 in-frame, and 5 fusion mutations) were detected, which were located between amino acids 0 and 652 (Figure 8C). Among them, E121R was the most frequent mutation site, with 23 truncating mutations (Figure 8C). In addition, based on the TISIDB database, we further analyzed the relationship between CD93 gene mutation and ICB treatment. It was found that there was no significant mutation difference between responders and non-responders (Supplementary Table S1).

### Association Between CD93 Expression and TMB, MSI, and ICB

**FIGURE 9:**
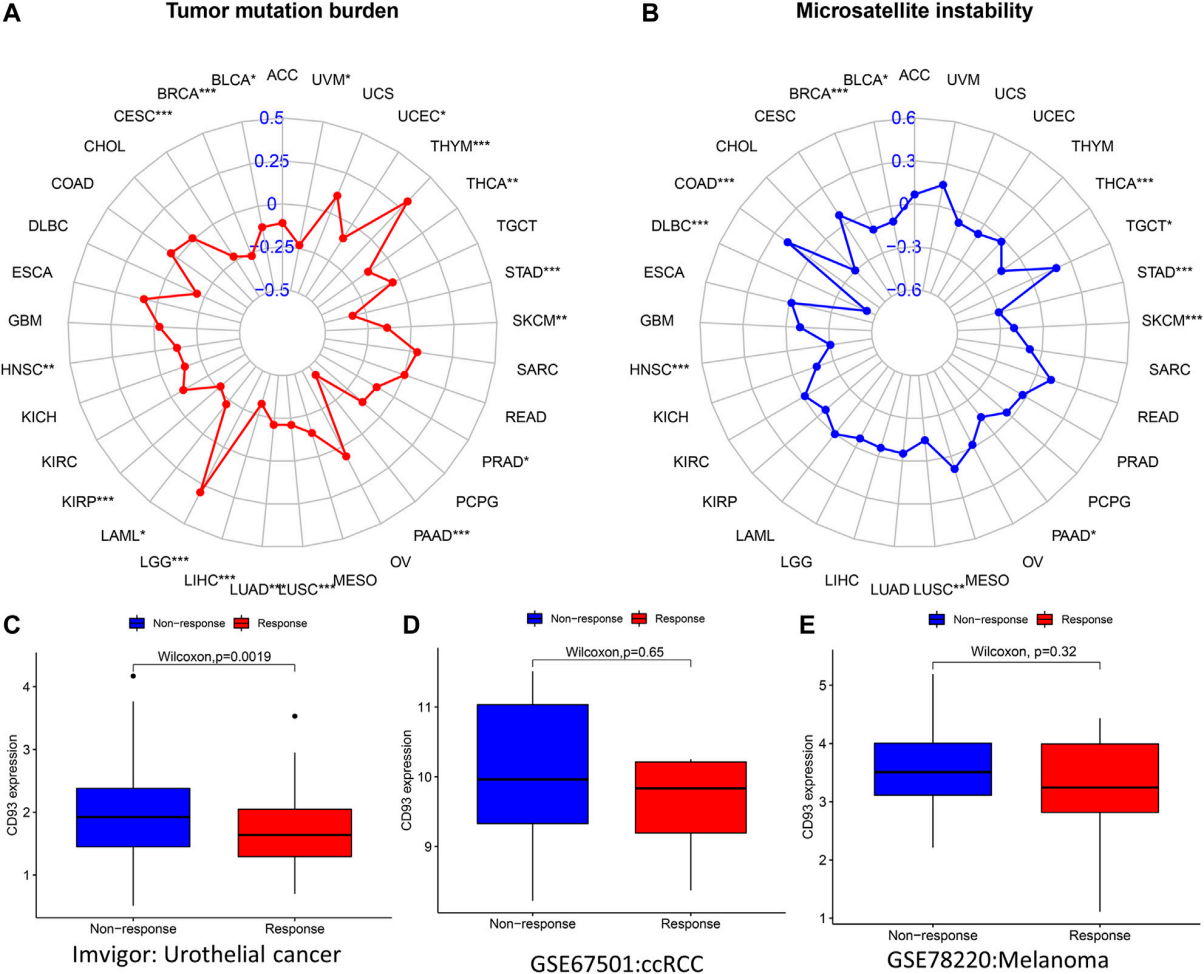
Correlations between CD93 expression and both immunotherapeutic markers and immunotherapeutic response. **(A,B)** Correlations between CD93 expression and tumor mutation burden (TMB) and microsatellite instability (MSI) in cancers. **(C–E)** Relationship between CD93 expression and response to immunotherapy in three cohorts (IMvigor, GSE67501, and GSE78220). *p < 0.05, *p* < 0.01, ***p < 0.001.

A previous study revealed that TMB was associated with tumorigenesis and is considered to be an independent predictor of the efficacy of immunotherapy (Samstein et al., 2019). MSI is the result of DNA mismatch repair (MMR) defects and is closely related to chemotherapy resistance and immunotherapy. Subsequently, we explored the relationship between CD93 expression and TMB and MSI. The expression of CD93 was positively associated with TMB in THYM and LGG (*p* < 0.001; Figure 9A), whereas it showed a negative correlation with TMB in the STAD, PAAD, LUSC, LIHC, LUAD, KIRP, CESC, and BRCA cohorts (*p* < 0.001; Figure 9A). For MSI, a positive association in the COAD cohort (*p* < 0.001; Figure 9B) and a negative association in the THCA, STAD, SKCM, HNSC, DLBC, and BRCA cohorts (*p* < 0.001; Figure 9B) were identified. As shown in Figure 9C (*p* < 0.01), patients with a low expression of CD93 were more sensitive to treatment with atezolizumab in urothelial cancer. However, no significant difference was found in the GSE67501 (*p* > 0.05; Figure 9D) and GSE78820 (*p* > 0.05; Figure 9E) cohorts. Using the TIMER2.0 database, we analyzed the co-expression relationship between CD93 and immune checkpoint genes (*CTLA-4* and *PD-L1*). The results showed that CD93 was co-expressed with *PD-L1* in COAD, BRCA, and LIHC (Supplementary Figures S3A–C). In addition, there was an obvious co-expression relationship between CD93 and *CTLA-4* in COAD and LUSC (Supplementary Figures S3D–E). Meanwhile, the expression level of CD93 in the mutated *POLE* (polymerase epsilon) group was significantly higher than that in the wild-type (WT) *POLE* group in COAD (Supplementary Figure S3F).

## Discussion

As a transmembrane glycoprotein, CD93 can be expressed in endothelial cells, stem cells, and bone marrow cells (Galvagni et al., 2017). Early studies have found that CD93 can promote the adhesion and penetration of immune cells. Recently, it has been reported that CD93 is a new type of angiogenic activator, mainly by promoting endothelial cell adhesion and accelerating tumor angiogenesis, thus affecting tumor growth. Its high expression can accelerate tumor growth and reduce host survival (Langenkamp et al., 2015). In addition, the outer domain of CD93 on the membrane surface can split or fall off easily under the stimulation of inflammation, forming soluble CD93 and promoting the process of asthma exacerbation (Sigari et al., 2016).

In this study, we analyzed the CD93 expression levels of 33 tumors in TCGA using the TIMER database. The results showed significant differences in the expression of CD93 between cancer tissues and normal tissues. Compared with normal tissues, its expression levels in CHOL, GBM, KIRC, LIHC, STAD, and THCA were significantly upregulated, while those in BLCA, BRCA, CESC, COAD, HNSC, KICH, KIRP, LUAD, LUSC, and UCEC were significantly downregulated. However, according to the IHC results from the HPA, except for liver, stomach, and pancreatic cancer, the CD93 protein was negative in most tumors. The expression of CD93 only existing in proliferative vascular endothelial cells may be one of the reasons for this result. The results of GSEA showed that, in COAD, BLCA, KIRC, and LIHC, the gene set of the CD93 high expression group was mainly enriched in the cell migration and tissue migration pathway, which is closely related to tumor angiogenesis.

Meanwhile, survival analysis based on pan-cancer showed that CD93 was a risk factor in most tumors and that an increased expression of CD93 usually indicated poor prognosis.

Furthermore, an increased expression of CD93 usually correlated with advanced TNM stage in multiple cancers, which was consistent with the results of the survival analysis. These results suggest that CD93 can be used as a robust prognostic biomarker. However, the OS, DSS, and PFS analyses showed that an elevated expression of CD93 was associated with better prognosis and lower TNM stage in KIRC, which was contrary to the results of other tumors. This suggests that CD93 may be a protective factor in KIRC, but the specific mechanism is worthy of further study.

In addition, we found that the expression of CD93 was significantly correlated with the level of immune infiltration in tumor tissues. Studies have shown that macrophages play an important role in tumorigenesis and metastasis. Under certain conditions, tumor-associated macrophages (TAMs) can differentiate into pro-inflammatory M1 and anti-inflammatory M2 macrophages (Shu and Cheng, 2020; Chen et al., 2021). M1 TAMs play an important role in the immune process of killing tumor cells, while M2 TAMs can promote tumor growth and invasion (Pan et al., 2020; Sa et al., 2020). In BLCA and TGCT, the expression of CD93 was positively correlated with the abundance of M2 macrophages. Therefore, there was a significantly positive correlation between a higher TNM stage and an increased expression of CD93 in TGCT and BLCA. Moreover, the prognosis of BLCA patients with a high expression of CD93 was worse than those with a low expression. In addition, the number of CD4^+^ T cells in KIRC were positively correlated with CD93 expression. A high expression of CD93 can recruit more CD4^+^ T cells and exert antitumor effect. Therefore, this may be one of the reasons for KIRC patients having high CD93 expression but with better prognosis. In addition, there was a significant positive correlation between neutrophil infiltration and CD93 expression in GBM. This is consistent with the conclusion of previous studies that CD93 was involved in the inflammatory process of the central nervous system (Griffiths et al., 2018).

In addition, we analyzed the relationship between the expression of CD93 and three immune modes (immune stimulators, immune inhibitors, and MHC molecules). In ACC and PCPG, the expression of CD93 was positively correlated with *TMEM173*. A previous study reported that ferroptotic damage promoted pancreatic tumorigenesis through a *TMEM173*/STING-dependent DNA sensor pathway (Dai et al., 2020). Meanwhile, CD93 and KDR have shown a significant co-expression relationship in various tumors. Current evidence shows that the expression of p-KDR is closely related to the density of vasculogenic mimicry (VM) in GBM and adverse clinical outcomes (Zaman et al., 2020).

Based on the TISIDB database, we further analyzed the relationship between the expression of CD93 and the immune subtypes of different cancer species. The results showed that there were significant differences in the expression of CD93 in different immune subtypes of multiple tumors. These results suggest that CD93 may be valuable as a marker for differentiating tumor immune subtypes, thus guiding clinical precision therapy.

More and more studies have demonstrated that genomic mutations participated in tumorigenesis, progression, and chemotherapy resistance (Gorelick et al., 2020). For example, a recent study has revealed that abnormal Rac1 activity and expression were closely related to tumor characteristics such as tumor genesis, survival, metastasis, anti-apoptosis, and drug resistance. Increased Rac1 activity or expression caused by gene mutation can promote the occurrence, development, metastasis, and invasion of tumors, resulting in the poor prognosis of patients (Lionarons et al., 2019). Li found that coding and non-coding mutations, as well as epimutations, converged on pathways that are important for prostate cancer (Li et al., 2020). In this study, we discovered that mutations of CD93 were most common in UCEC (>7%), followed by STAD, COAD, and SKCM. However, we found no significant associations between CD93 mutations and immunotherapy response.

The current study revealed that a high TMB can benefit immunotherapy for multiple tumor types (Pérez-Ruiz et al., 2020). MSI is a mutation caused by a defective DNA mismatch repair and is a potential predictive marker for immunotherapy (Wu et al., 2021). In this study, we further analyzed the relationship between CD93 expression and TMB and MSI. There was a significant positive correlation between CD93 expression and TMB, especially in LGG and THYM. Meanwhile, the expression of CD93 was negatively correlated with MSI, especially in DLBC. Previous studies indicated that an increased expression of CD93 can promote vascular endothelial cell migration and vascular maturation in tumor tissue, also leading to T-cell depletion (Sun et al., 2021). Blocking CD93 can increase the abundance of T cells in the tumor microenvironment and improve the sensitivity of patients to immunotherapy (American Association for Cancer Research, 2021; Sun et al., 2021). Our study revealed that patients with a low expression of CD93 were more suitable for immunotherapy in the cohort of urothelial cancer. Therefore, CD93 has potential value as a biomarker of immunotherapy in urothelial cancer.

There are some limitations to this study. Firstly, further experiments are needed to determine the precise molecular function of CD93 in tumorigenesis. For example, RT-PCR, Western blot, IHC, and other experimental methods can be used to verify the expression of CD93 in pan-cancer. Secondly, in some tumor types, the sample size in the TCGA database was limited, which may lead to some bias in the analysis results. Thirdly, more external datasets are needed to verify the relationship between CD93 and immunotherapy.

## Conclusion

We used integrated bioinformatics approaches to show that the expression of CD93 was closely related to the tumor stage and immune infiltration of pan-cancer and affected the prognosis of patients, so it has potential value to be a biomarker of prognosis. CD93 was highly involved in various immune responses, especially in urothelial cancer. Therefore, CD93 blockade combined with existing checkpoint inhibitors may be a feasible way to inhibit the progress of urothelial cancer. The development of immune checkpoint inhibitors against CD93 is expected to play an important role in the immunotherapy of malignant tumors.

## Data Availability

The datasets presented in this study can be found in online repositories. The names of the repository/repositories and accession number(s) can be found in the article/Supplementary Material.

## Abbreviations

OS, overall survival; DFS, disease-free survival; DSS, disease-specific survival; PFS, progression-free survival; TCGA, The Cancer Genome Atlas; GO, Gene Ontology; TAMs, tumor-associated macrophages.
