# Supplementary figures and images for: Pan-Cancer Analysis Identified CD93 as a Valuable Biomarker for Predicting Patient Prognosis and Immunotherapy Response
Source: Front Mol Biosci. 2022 Feb 21;8:793445. doi: 10.3389/fmolb.2021.793445 (PMC8900912; doi:10.3389/fmolb.2021.793445)

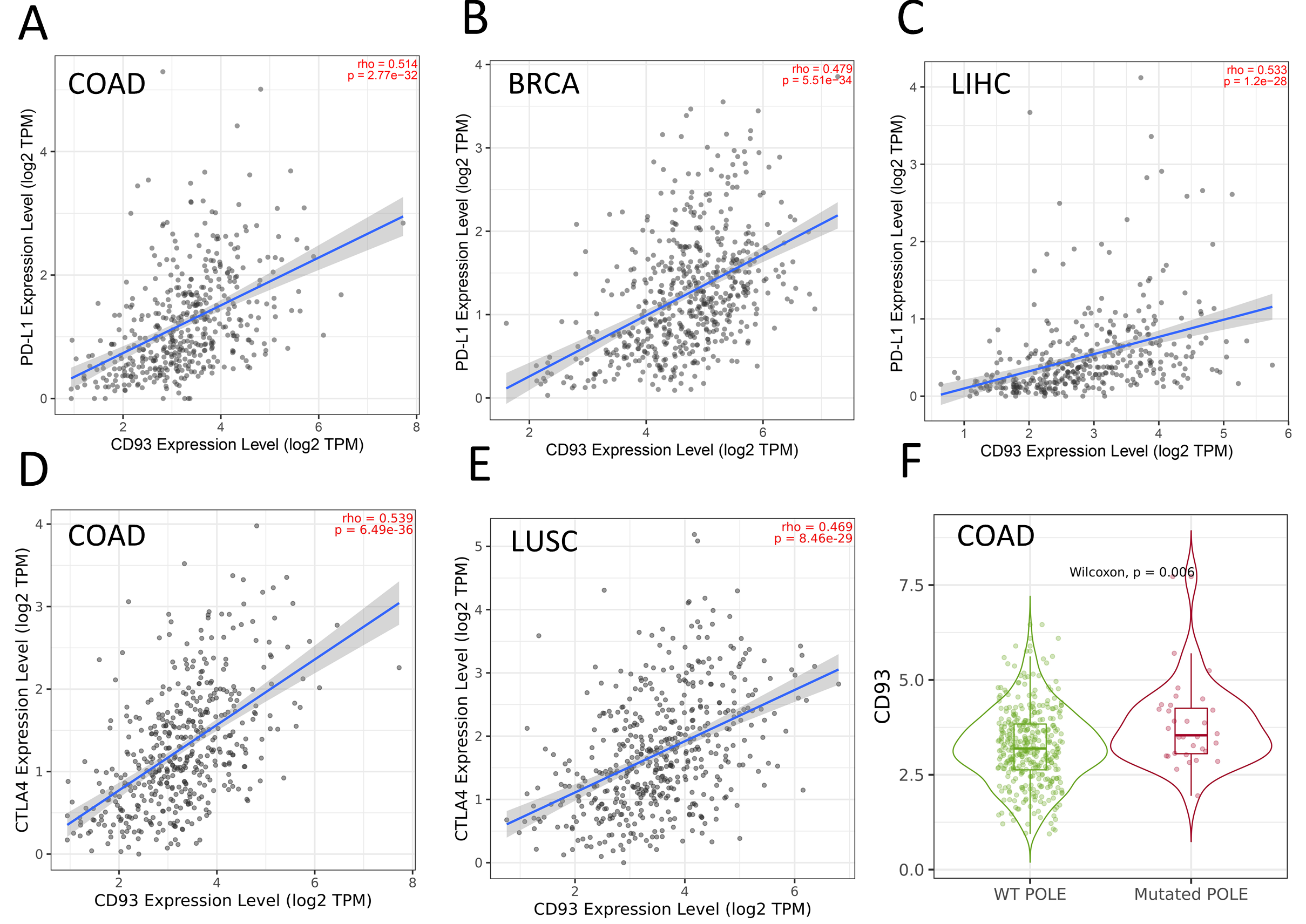

Supplement: Supplementary file 1 [file Image3.TIF]

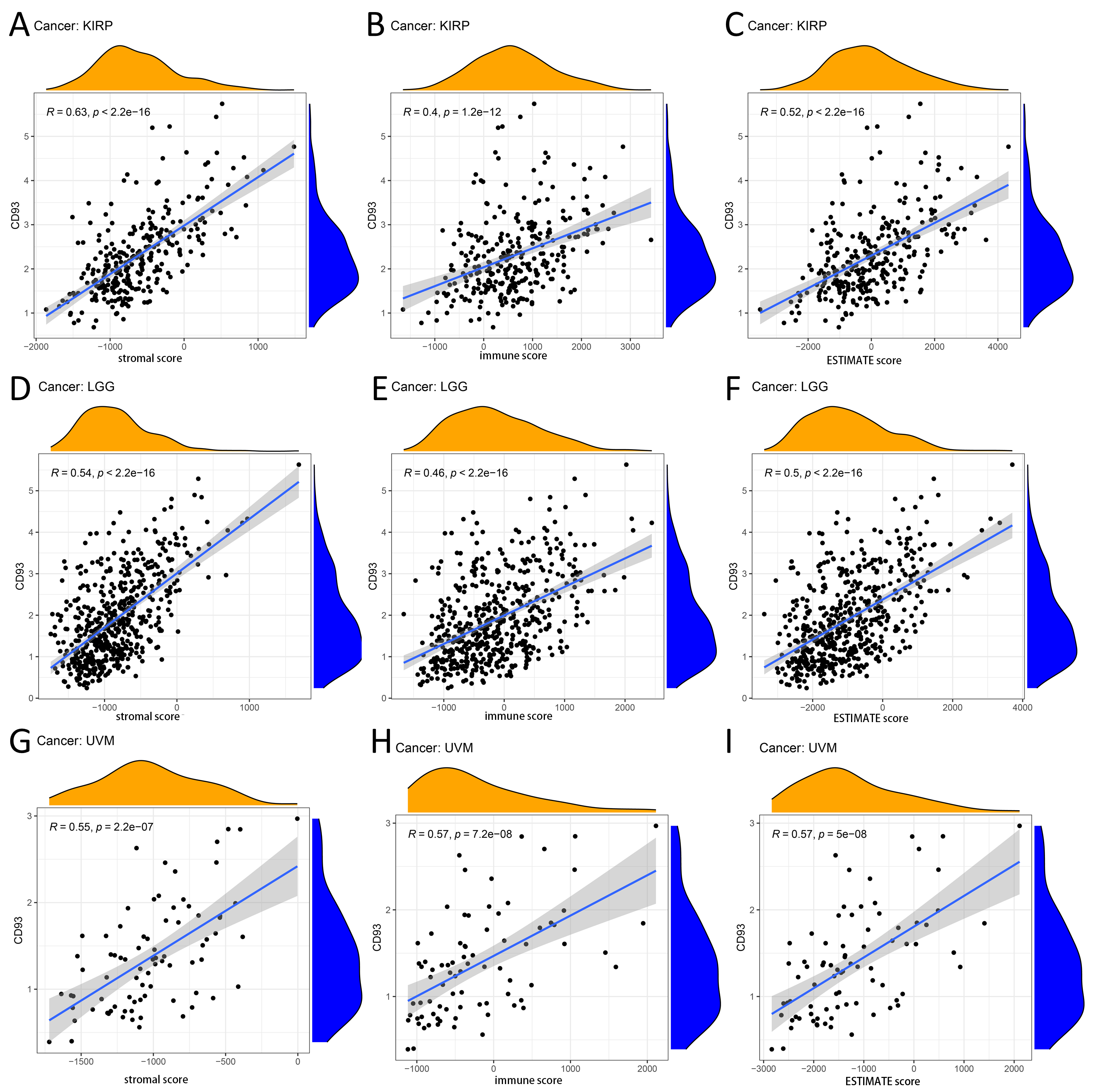

Supplement: Supplementary file 2 [file Image2.TIF]

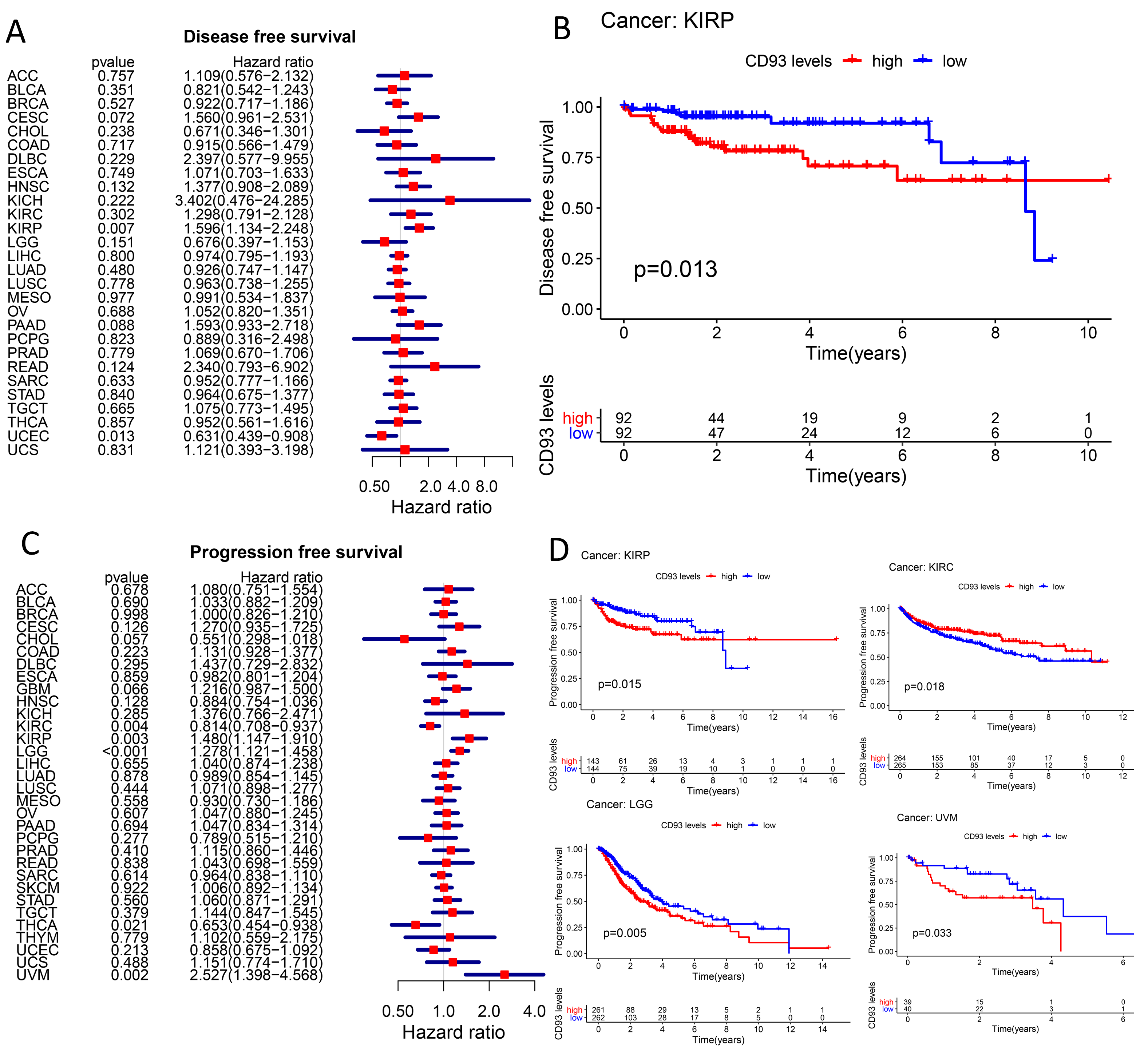

Supplement: Supplementary file 3 [file Image1.TIF]
